# Allogeneic hematopoietic cell transplantation with reduced-toxicity conditioning for X-linked hyper-IgM syndrome

**DOI:** 10.1007/s12185-026-04245-8

**Published:** 2026-07-07

**Authors:** Ryosuke Wakatsuki, Satoshi Miyamoto, Akihiro Hoshino, Shuhei Takahashi, Yusuke Ishibashi, Ryohei Watanabe, Tatsuya Magara, Dan Tomomasa, Akira Nishimura, Kyogo Suzuki, Takahiro Kamiya, Takeshi Isoda, Kohsuke Imai, Michiko Kajiwara, Tomohiro Morio, Masatoshi Takagi, Hirokazu Kanegane

**Affiliations:** 1 Department of Pediatrics and Developmental Biology, Graduate School of Medical and Dental Sciences, Institute of Science Tokyo, Tokyo, Japan; 2 Department of Child Health and Development, Graduate School of Medical and Dental Sciences, Institute of Science Tokyo, 1-5-45 Yushima, Bunkyo-ku, Tokyo 113-8519 Japan; 3 Department of Pediatrics, National Defense Medical College, Saitama, Japan; 4 Center for Transfusion Medicine and Cell Therapy, Institute of Science Tokyo Hospital, Tokyo, Japan; 5 Laboratory of Immunology and Molecular Medicine, Institute of Science Tokyo, Tokyo, Japan

**Keywords:** X-linked hyper-IgM syndrome, Inborn errors of immunity, Hematopoietic cell transplantation, Reduced-toxicity conditioning, Graft-versus-host disease

## Abstract

X-linked hyper-IgM syndrome (XHIGM) is an inborn error of immunity caused by variants in the *CD40LG* gene, leading to disrupted T-cell function and impaired immunoglobulin class-switch recombination. Supportive care alone is linked to unfavorable outcomes, necessitating allogeneic hematopoietic cell transplantation (HCT) as a curative option. Although myeloablative conditioning is recommended for HCT, concerns remain regarding its toxicity. We reviewed ten patients with XHIGM who underwent HCT using reduced-toxicity conditioning (RTC) at our institution between 2010 and 2024. All patients underwent HCT by 5 years of age (range: 2–5 years; median: 3 years). Donor sources included unrelated bone marrow, unrelated cord blood, and haploidentical-related donors. Conditioning primarily consisted of fludarabine and busulfan (target area under the curve: 60–65 mg/L·h), with low-dose total body irradiation (TBI) administered in five patients. Two patients experienced graft failure, requiring a second transplantation, and one died from post-transplant complications. The remaining nine patients survived. The 5-year overall survival rate was 88.9%; the 5-year graft-versus-host disease-free, relapse-free, and second transplantation-free survival rate was 67.5%. The addition of low-dose TBI to fludarabine/busulfan was associated with favorable long-term donor chimerism, although this finding should be considered hypothesis-generating. RTC may represent a feasible conditioning strategy for XHIGM.

**Supplementary Information:**

The online version contains supplementary material available at https://doi.org/10.1007/s12185-026-04245-8.

## Introduction

X-linked hyper-IgM syndrome (XHIGM), also known as CD40L deficiency, is a rare and severe inborn error of immunity (IEI) caused by hemizygous variants in the *CD40LG* gene on the X chromosome [1–5]. Its estimated prevalence is approximately one per 1,000,000 individuals [6]. XHIGM is characterized by an absent interaction between CD40L on activated CD4^+^ T cells and CD40 on antigen-presenting cells, including B cells, dendritic cells, and macrophages. In B cells, this leads to impaired class-switch recombination and somatic hypermutation, markedly reducing serum IgG, IgA, and IgE levels, whereas IgM levels remain normal or elevated. Moreover, CD40L-CD40 signaling is also essential for effective T-cell activation through interactions with antigen-presenting cells [7–9]. Consequently, XHIGM is classified as a combined immunodeficiency (CID) [10].

Patients with XHIGM typically present in infancy with recurrent pneumonia and otitis media. Furthermore, characteristic manifestations include interstitial pneumonia caused by *Pneumocystis jirovecii* (PJP), observed in up to 40% of patients, as well as chronic diarrhea due to *Cryptosporidium* infection. Additional complications include neutropenia, sclerosing cholangitis, and malignancy [11]. Although supportive care, including immunoglobulin replacement therapy (IgRT) and prophylactic trimethoprim-sulfamethoxazole and antifungal agents, provides clinical benefit, it alone is associated with a survival rate of only 28.2% at 40 years of age [11, 12]. Currently, allogeneic hematopoietic cell transplantation (HCT) is the only curative option for XHIGM. HCT is recommended relatively early, generally before 5–10 years of age, because complications such as pre-transplant sclerosing cholangitis, chronic lung disease, *Cryptosporidium* infection, and prolonged diarrhea impact prognosis [12, 13].

Regarding conditioning intensity, the 2021 EBMT/ESID Inborn Errors Working Party guidelines recommend myeloablative conditioning (MAC) for HCT in IEIs classified as CIDs, such as XHIGM [14]. Similarly, a European international collaborative study published in 2019 summarizing HCT outcomes in XHIGM supported the use of MAC with a fludarabine (FLU)-plus busulfan (BU: 14.3–25.0 mg/kg)-based regimen [13]. Conversely, reduced-intensity conditioning (RIC) has been associated with an increased risk of post-transplant graft failure. However, MAC-based HCT may elevate the risk of late toxicities, thus establishing a more optimal conditioning intensity for XHIGM. In this study, we present our single-center experience with reduced-toxicity conditioning (RTC) in patients with XHIGM undergoing HCT. RTC was defined as an intermediate-intensity approach between MAC and RIC that incorporates additional conditioning agents and/or low-dose total body irradiation into RIC-based regimens.

## Materials and methods

### Patients

We retrospectively analyzed 10 patients with XHIGM who underwent HCT at the Institute of Science Tokyo (formerly Tokyo Medical and Dental University) Hospital over a 15-year period from 2010 to 2024 (Table 1). Follow-up data were collected through March 31, 2026. The clinical courses of patients 1–3 have been reported previously [15]. Two patients (patients 1 and 3) underwent a second transplantation owing to graft failure. In patient 1, the first HCT was performed at another institution, whereas the second was performed at our institution. For patient 3, the first HCT was performed at our institution, while the second was performed at another institution [15, 16].

**Table 1:** Characteristics of patients

	Age at diagnosis	*CD40LG* variant	Pre-transplant complications	*Cryptosporidium*
Patient 1	1 y	c.143delG, p.Arg48LysfsTer6	Aspergillosis (resolved)	NT
Patient 2	At birth	c.676G > A, p.Gly226Arg	–	NT
Patient 3	2 y	c.464 T > C, p.Leu155Pro	EBV/CMV past infection	Negative
Patient 4	At birth	c.510_511 ins A, p.Ile171AsnFsTer30	–	NT
Patient 5	4 m	c.107 T > G, p.Met36Arg	Pneumocystis pneumonia and CMV viremia (resolved)	Negative
Patient 6	10 m	c.594_595insG, p.Arg200Ter	Pneumocystis pneumonia (resolved)	NT
Patient 7	1 y	c.330delC, p.Phe111LeuFsTer17	Nonspecific chronic colitis (resolved)	NT
Patient 8	At birth	c.330delC, p.Phe111LeuFsTer17	Aspergillus antigen positive	NT
Patient 9	At birth	c.674 T > C, p.Leu225Ser	–	NT
Patient 10	4 y	c.507 T > G, p.Tyr169Ter	Aspergillus antigen positive	Negative

y: year(s), m: months, EBV: Epstein–Barr virus, CMV: cytomegalovirus, NT: not tested

### Definition of engraftment and acute graft-versus-host disease

Neutrophil engraftment was defined as the first of 3 consecutive days on which the absolute neutrophil count was ≥ 0.5 × 10^9^/L following HCT. Acute graft-versus-host disease (GVHD) was graded according to the modified Glucksberg criteria. Post-transplant hemophagocytic lymphohistiocytosis (PT-HLH) was diagnosed according to the criteria proposed by Asano et al. [17], which include fever, cytopenia, elevated ferritin (> 500 ng/mL) and lactate dehydrogenase levels, and hemophagocytosis in the bone marrow. Graft failure was clinically determined by the treating physicians based on serial donor chimerism, hematopoietic recovery, clinical course, and the need for second transplantation; persistent mixed chimerism alone was not considered graft failure.

### Chimerism analysis

Donor chimerism following transplantation was assessed using various methods selected according to donor source and case characteristics. Short tandem repeat (STR) analysis was used to evaluate the first transplantation in patient 1 as well as transplants in patients 3, 5, 6, and 7. The second transplantation in patient 1 and the transplantation in patient 4 were assessed using sex-mismatched fluorescence in situ hybridization (FISH). Patients 2 and 6 were evaluated by HLA-based flow cytometry (HLA-flow) (Supplementary Fig. 1) [18]. In patient 8, sex-mismatched droplet digital polymerase chain reaction (ddPCR) was performed targeting *SRY*, whereas patients 9 and 10 were assessed by variant-specific ddPCR (Supplementary Fig. 2) [19, 20]. For STR analysis, values below the detection limit of 5% were considered 0% chimerism, whereas values ≥ 95% were considered 100% chimerism for statistical analysis.

### Outcomes

Overall survival (OS) and GVHD-free, relapse-free, and second transplantation-free survival (GRSFS) were calculated from the date of the first transplantation (day 0) [21]. OS was defined as the time from the first transplantation to death from any cause or last follow-up; patients were not censored at second transplantation. GRSFS was defined as the time from the first transplantation to the onset of grade III or higher GVHD or to graft failure requiring second transplantation, whichever occurred first. Procedure-related complications, including acute GVHD and PT-HLH, were summarized on a transplantation-procedure basis.

### Statistical analysis

OS and GRSFS were estimated using the Kaplan–Meier method. Chimerism was visualized using spaghetti plots depicting the post-transplant days and the percentage of donor chimerism in whole blood for each patient. For procedure-based summaries and chimerism plots, the first HCTs in patients 1 and 3 were designated as patient 1–1 and 3–1, respectively, and the second HCTs as patients 1–2 and 3–2, respectively. OS and GRSFS were analyzed using GraphPad Prism 11 (GraphPad, San Diego, CA, USA), and spaghetti plots were generated using R version 4.5.2 (R Core Team, Vienna, Austria) and RStudio IDE version 2026.01.1 (Posit Software, PBC, Boston, MA, USA).

## Results

### Patient characteristics and transplantation details

Most patients in this study population were diagnosed with XHIGM at birth or during infancy/early childhood (range: birth to 4 years; median: 11 ± 15.2 months), and all underwent HCT by 5 years of age (range: 2–5 years; median: 3 years). Aspergillosis was identified in three patients (patients 1, 8, and 10) prior to transplantation; however, infections were well controlled in all patients at the time of transplantation. Two patients (patients 5 and 6) developed PJP before or at diagnosis; however, both underwent transplantation following recovery. Stool testing for *Cryptosporidium* was performed in three patients (patients 3, 5, and 10), yielding negative results in these cases. In patient 7, nonspecific chronic colitis accompanied by hypoalbuminemia and hypogammaglobulinemia was reported. However, these abnormalities promptly improved after initiation of IgRT following the diagnosis of XHIGM, and HCT was subsequently performed in the absence of active enterocolitis. Additionally, no symptoms of chronic diarrhea were observed before HCT in patients who were not screened for *Cryptosporidium*. Moreover, no patient developed sclerosing cholangitis.

For the transplantation, the donor sources included unrelated bone marrow in six patients (patients 1, 3, 4, 5, 8, and 10), unrelated cord blood in two patients (patients 1–2 and 2), and haploidentical-related donors in four patients (patients 3–2, 6, 7, and 9). Conditioning was primarily based on FLU and BU, with a target BU area under the curve (AUC) of 60–65 mg·h/L. Seven patients (patients 1–2, 3, 6, 7, 8, 9, and 10) received serotherapy with alemtuzumab or rabbit anti-thymocyte globulin (ATG), and five patients (patients 4, 5, 6, 7, and 10) also received low-dose total body irradiation (TBI). For GVHD prophylaxis, patients undergoing haploidentical transplantation (patients 3–2, 6, 7, and 9) were administered post-transplant cyclophosphamide (PT-CY), tacrolimus, and mycophenolate mofetil (MMF), whereas the other patients received tacrolimus plus short-course methotrexate (Table 2).

**Table 2:** Summary of allegeneic hematopoietic cell transplantation for X-linked hyper-IgM syndrome

	Age at transplantation	Donor source	HLAmatch	Pre-transplant conditioning	BU AUC (mg/L·h)	Intensity of pre-transplant preparation	GVHD prophylaxis	Engraftment (days)	Acute GVHD	Infections and other complications	HLH(days to onset)	Outcome
Patient 1	4	URBMT	6/8	BU 16 mg/kg + CY 200 mg/kg	no data	MAC	Tac + short MTX	19	Grade I Skin stage 2	–	21	graft failure
Patient 1–2	5	CBT	6/8	rabbit ATG 2 mg/kg + Flu180 mg/m^2^ + BU 16 mg/kg	no data	RIC	Tac + short MTX	46	Grade I Skin stage 1	–	35	Alive
Patient 2	2	CBT	7/8	Flu 180 mg/m2 + BU 14.3 mg/kg	64.91	RIC	Ta c + short MTX	25	Grade II Skin stage 3	CMV viremia	–	Alive
Patient 3	3	URBMT	7/8	rabbit ATG 2.5 mg/kg + Flu 180 mg/m^2^ + BU 16 mg/kg	61.23	RIC	Tac + short MTX	28	–	BKV viremia, CMV viremia	26	graft failure
Patient 3–2	3	haplo-BMT	4/8	rabbit ATG 2.5 mg/kg + Flu 180 mg/m^2^ + Mel 210 mg/m^2^ + Eto 200 mg/m^2^		MAC	Tac + PT-CY + MMF		–	TMA gastrointestinal bleeding,	–	death on post-transplant day 65
Patient 4	2	URBMT	8/8	Flu 180 mg/m^2^ + BU 14.5 mg/kg + TBI 3 Gy	61.8	RTC	Tac + short MTX	35	–	–	–	Alive
Patient 5	2	URBMT	7/8	Flu 180 mg/m^2^ + BU 11.2 mg/kg + TBI 3 Gy	65	RTC	Tac + short MTX	22	–	TMA	13	Alive
Patient 6	4	haplo-BMT	4/8	rabbit ATG 2.5 mg/kg + Flu 180 mg/m^2^ + BU 16.6 mg/kg + TBI 3 Gy	65.9	RTC	Tac + PT-CY + MMF	15	–	–	–	Alive
Patient 7	4	haplo-BMT	4/8	rabbit ATG 2.5 mg/kg + Flu 180 mg/m^2^ + BU 11.0 mg/kg + TBI 3 Gy	65.2	RTC	Tac + PT-CY + MMF	17	Grade Ⅲ GI stage 2 Skin stage 3	hemorrhagic cystitis(BKV-associated)	–	Alive
Patient 8	3	URBMT	8/8	ALZ 0.64 mg/kg + Flu 180 mg/m^2^ + BU 12.9 mg/kg	65	RIC	Tac + short MTX	14	Grade Ⅰ Skin stage 1	–	–	Alive
Patient 9	3	haplo-BMT (Father)	5/8	ALZ 0.64 mg/kg + Flu 180 mg/m^2^ + BU 17.2 mg/kg	64.6	RIC	Tac + PT-CY + MMF	16	–	CMV viremia	–	Alive
Patient 10	5	URBMT	7/8	rabbit ATG 2.5 mg/kg + Flu 180 mg/m^2^ + BU 18.9 mg/kg + TBI 2 Gy	66.8	RTC	Tac + short MTX	26	Grade Ⅱ Skin stage 3	CMV viremia, EBV viremia	90	Alive

URBMT: unrelated bone marrow transplantation, CBT: cord blood transplantation, HLA: human leukocyte antigen, ATG: anti-thymocyte globulin, Mel: melphalan, Eto: etoposide, ALZ: alemtuzumab, TBI: total body irradiation, AUC: area under the curve, MAC: myeloablative conditioning, RIC: reduced-intensity conditioning, RTC: reduced-toxicity conditioning, GVHD: graft-versus-host disease, Tac: tacrolimus, PT-CY: post-transplant cyclophosphamide, MMF: mycophenolate mofetil, BKV: BK virus, TMA: thrombotic microangiopathy, HLH: hemophagocytic lymphohistiocytosis

### Transplant outcomes

**Fig. 1:**
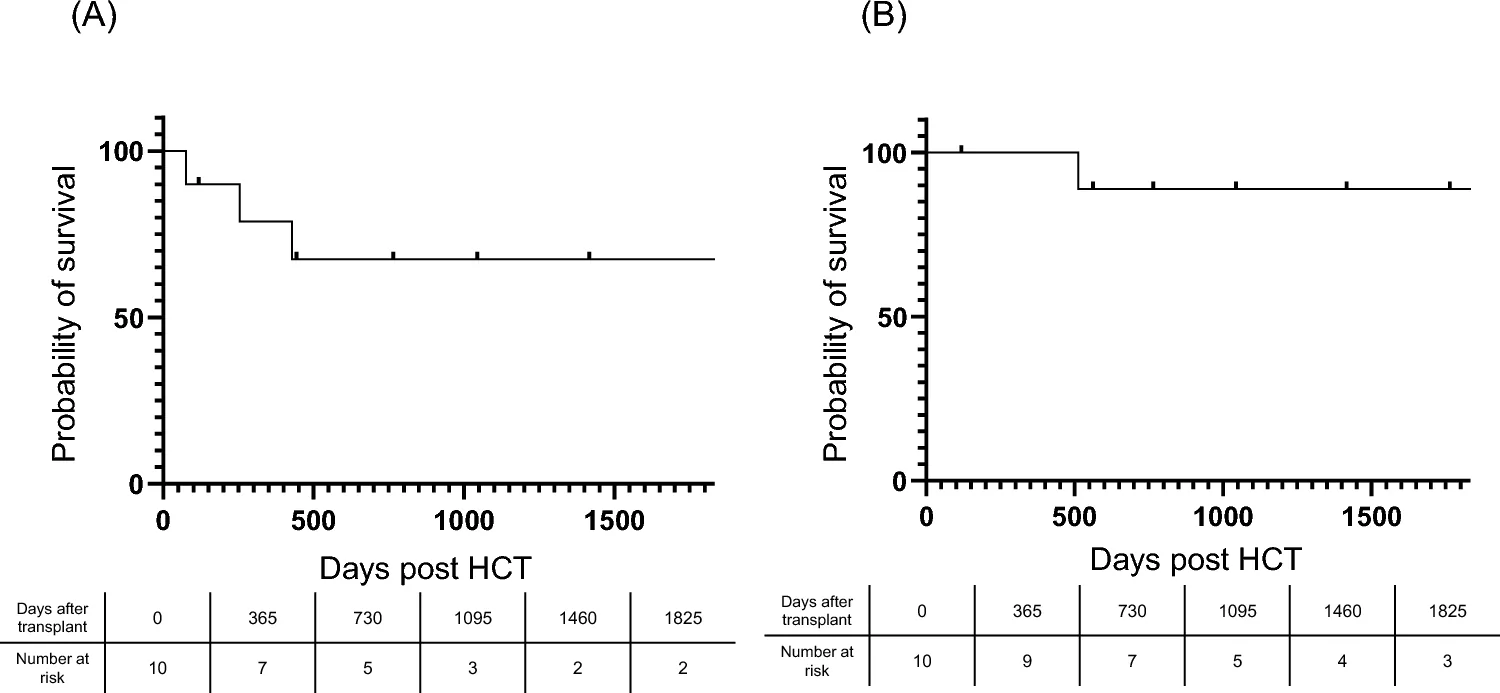
Transplant outcomes in 10 patients with X-linked hyper-IgM syndrome. **A** Kaplan–Meier curve showing 5-year graft-versus-host disease (GVHD)-free, relapse-free, and second transplantation-free survival (GRSFS) following transplantation. GRSFS was defined using the onset of grade III or higher GVHD or the date of the second transplantation as endpoints. **B** Kaplan–Meier curve showing 5-year overall survival (OS) following transplantation. OS was defined as the time from transplantation to death from any cause Numbers at risk are shown below each Kaplan–Meier curve.

The median follow-up duration after transplantation was 1229.5 days (range: 118–5484 days). The median time to neutrophil engraftment was 20.5 days (range: 14–35 days). Patients 1 and 3 developed graft failure following the first HCT, necessitating a second transplantation. Patient 1 developed PT-HLH after the first HCT and was clinically diagnosed with graft failure on day 254, when chimerism analysis showed near-complete recipient-type hematopoiesis; the interval between the first and second HCTs was 534 days. Patient 3 developed severe aplastic anemia after the first HCT and was clinically diagnosed with graft failure on day 428, when chimerism analysis demonstrated near-complete recipient-type hematopoiesis; the interval between the first and second HCTs was 497 days. Patient 7 developed grade III acute GVHD during follow-up, with stage II skin and gastrointestinal involvement. Moreover, the 5-year GRSFS was 67.5% (95% CI, 29.1–88.3; Fig. 1A**, **Table 2).

Skin GVHD occurred in five of 10 patients and after six of 12 transplantation procedures (50%; patients 1, 2, 7, 8, and 10, including both transplantations in patient 1) and improved in all cases following treatment with corticosteroids and/or JAK inhibitors. Grades II-IV acute GVHD occurred after three of 12 transplantation procedures (25%; patients 2, 7, and 10). PT-HLH developed in four of 10 patients and after five of 12 transplantation procedures (42%; patients 1, 3, 5, and 10, including both transplantations in patient 1); most cases were diagnosed clinically and responded promptly to dexamethasone palmitate. Patient 3 died of gastrointestinal bleeding due to thrombotic microangiopathy 65 days after the second transplantation. The remaining nine patients survived without chronic complications. Of the nine surviving patients, the clinical course of immunoglobulin replacement therapy was evaluated in eight patients. Seven of these eight patients discontinued IgRT, while patient 8 continued to require IgRT throughout the observation period. The median time to discontinuation of IgRT was 12 months after HCT (Supplementary Fig. 3).

The 5-year OS was 88.9% (95% CI, 43.3–98.4; Fig. 1B**, **Table 2).

### Chimerism

**Fig. 2:**
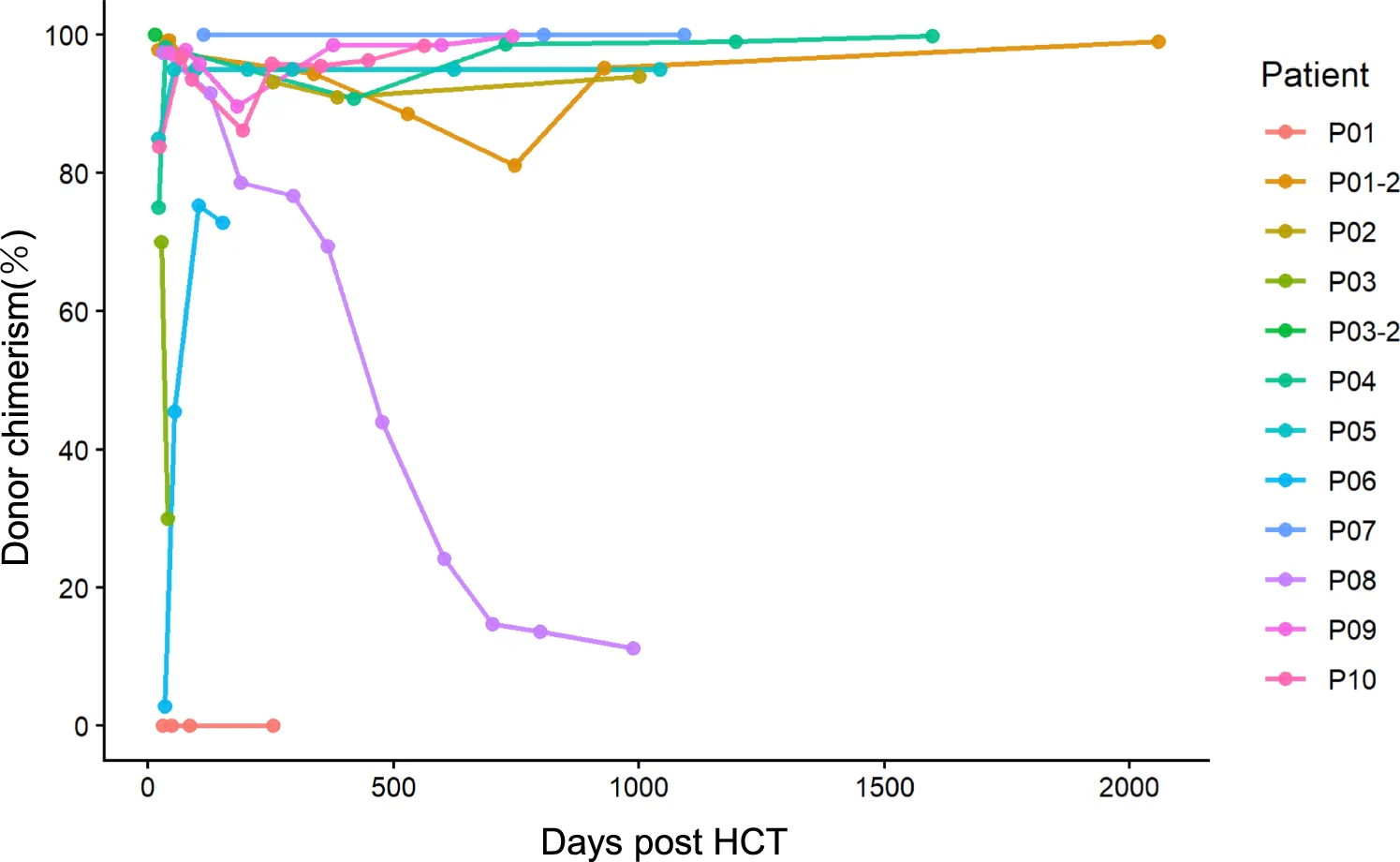
Changes in donor chimerism over time. Spaghetti plot illustrates the percentage of donor chimerism in whole blood over time following transplantation for each patient with XHIGM. For patients 1 and 3, who underwent a second transplantation, the first hematopoietic cell transplantation (HCT) was labeled as 1–1 and 3–1, respectively, and the second HCT was labeled as patient 1–2 and 3–2, respectively

Donor chimerism was generally favorable, including long-term maintenance, for the majority of patients (Fig. 2). However, graft failure after the first HCT occurred in patients 1 and 3, whereas patient 8 showed progressive graft loss with persistent mixed chimerism but had not been clinically diagnosed with graft failure at the last follow-up. In patient 8, the most recent donor T-cell chimerism was 25.2% (Supplementary Fig. 4). Conversely, all five patients (patients 4, 5, 6, 7, and 10) who received low-dose TBI in addition to FLU + BU achieved sustained engraftment without graft failure and demonstrated more favorable long-term chimerism. Notably, the incidence of acute GVHD was similar between the TBI and non-TBI groups (2/5 [40%] vs. 3/5 [60%], respectively).

## Discussion

Previous research has suggested that RIC increases the risk of graft failure in HCT for XHIGM [13]. Accordingly, the EBMT/ESID Inborn Errors Working Party guidelines recommend MAC for CIDs such as XHIGM [14]. Recommended regimens include BU (AUC 85–95 mg·h/L) plus FLU (160 mg/m^2^) or treosulfan (30–42 g/m^2^) plus FLU (150–160 mg/m^2^) plus thiotepa (8–10 mg/kg). Although MAC reduces the risk of graft failure, it may increase late toxicities.

In this study, all 10 patients underwent HCT by the age of 5 years and were diagnosed relatively early, enabling transplantation under optimal supportive care before the development of major irreversible complications. Both the 5-year OS and GRSFS appeared broadly comparable to those reported in the post-2000 cohort of the 2019 European Collaborative Study [13]. However, this historical comparison should be interpreted cautiously because of differences in treatment era, patient selection, and sample size.

When long-term chimerism was considered, the five patients who received rabbit ATG (2.5 mg/kg), FLU (180 mg/m^2^), BU with an AUC of 60–67 mg·h/L, and additional low-dose TBI (2–3 Gy) demonstrated favorable outcomes without a discernible increase in complications. Conversely, patients 3 and 8, who received FLU 180 mg/m^2^ plus BU (AUC 61 or 65 mg·h/L) without TBI, experienced graft failure and progressive graft loss with persistent mixed chimerism, respectively, suggesting that this RIC regimen alone may be insufficient for XHIGM. Although patient 8 has not met the criteria for graft failure and remains clinically stable, declining donor chimerism suggests that second transplantation may eventually become necessary. Collectively, our findings suggest that FLU 180 mg/m^2^ plus BU at an AUC of 60–65 mg·h/L alone may be insufficient for XHIGM and that modest intensification of this RIC backbone may be preferable. In our cohort, this intensification was achieved by adding low-dose TBI. Although interpretation is limited by the small sample size and potential confounding arising from differences in donor source, GVHD prophylaxis, serotherapy, and transplant era, the favorable long-term chimerism observed in the low-dose TBI group suggests that this degree of conditioning intensity may have been appropriate for XHIGM. Furthermore, PT-CY was used in four cases of haploidentical transplants and may have influenced both engraftment and the incidence of GVHD. In particular, although patient 9 received the same conditioning regimen as patient 8, the use of PT-CY may have contributed to sustained engraftment and the prevention of GVHD in patient 9. Nevertheless, these observations should be considered hypothesis-generating.

The target BU AUC of 60–65 mg·h/L was chosen because this exposure corresponds to RIC protocol C defined in the EBMT/ESID Inborn Errors Working Party guidelines. We used this RIC-based BU exposure as the backbone of our regimen to maintain a reduced-toxicity profile rather than adopting the standard MAC target of 85–95 mg·h/L [14]. However, given the reported risk of graft failure after RIC in XHIGM, we considered that this backbone might require additional intensification to ensure stable long-term engraftment. In our cohort, this intensification was achieved by adding low-dose TBI, although our findings do not indicate that TBI itself is indispensable. Rather, the results suggest that when a RIC backbone is used in XHIGM, a certain degree of additional pretreatment intensity may be necessary to balance reduced toxicity with sustained donor chimerism.

In our cohort, low-dose TBI was the selected approach for modestly intensifying the RIC backbone and may have contributed to stable engraftment through both enhanced immunosuppression and improved marrow niching. By promoting lymphodepletion, low-dose TBI may have augmented the immunosuppressive intensity of the regimen, which may be particularly relevant in XHIGM, a CID in which potential residual host may contribute to graft rejection. In addition, low-dose TBI may have remodeled the bone marrow microenvironment. Because bone marrow macrophages are integrated within hematopoietic stem cell (HSC) niches and regulate HSC retention, mobilization, and engraftment, modulation of macrophage-dependent niche signals may have supported durable donor hematopoiesis [22].

This study had several limitations that should be acknowledged. First, this was a single-center retrospective study with a limited sample size of 10 patients. Therefore, conclusions regarding transplant outcomes and complications should be interpreted with caution and may differ in larger cohorts. Second, RTC in this study was achieved by adding low-dose TBI to an RIC backbone. In the present analysis, none of the five patients who received low-dose TBI developed TBI-related complications, such as growth impairment or endocrine dysfunction [23, 24]. Although low-dose TBI is associated with limited toxicity, it is not a radiation-free approach. Accordingly, we were unable to determine whether low-dose TBI was the optimal strategy for intensifying conditioning in XHIGM, whether increasing BU exposure would achieve better clinical outcomes, or what dose of treosulfan was the most appropriate. As novel agents and transplantation approaches continue to be developed, further disease-specific studies are required to optimize HCT strategies for IEIs including XHIGM.

## Conclusion

We retrospectively analyzed the clinical outcomes of 10 patients with XHIGM who underwent HCT at a single center over a 15-year period. Early diagnosis and appropriate supportive care enabled transplantation before the age of 5 years without major pre-transplant complications. Overall, our findings suggest that RTC may serve as a favorable conditioning strategy for XHIGM, offering a reasonable balance between transplant-related toxicity and durable long-term donor chimerism.

## Supplementary Information

Below is the link to the electronic supplementary material.

Supplementary file1 (DOCX 881 KB)

## Data Availability

The data supporting the findings of this study are available from the corresponding author upon reasonable request.

## Abbreviations

ATG: Anti-thymocyte globulin
AUC: Area under the curve
BU: Busulfan
CID: Combined immunodeficiency
ddPCR: Droplet digital polymerase chain reaction
FISH: Fluorescence in situ hybridization
FLU: Fludarabine
GRSFS: Graft-versus-host disease-free, relapse-free, and secondary graft failure-free survival
GVHD: Graft-versus-host disease
HCT: Hematopoietic cell transplantation
HSC: Hematopoietic stem cell
IEI: Inborn errors of immunity
IgRT: Immunoglobulin replacement therapy
MAC: Myeloablative conditioning
MMF: Mycophenolate mofetil
OS: Overall survival
PJP: *Pneumocystis jirovecii* pneumonia
PT-CY: Post-transplant cyclophosphamide
PT-HLH: Post-transplant hemophagocytic lymphohistiocytosis
RIC: Reduced-intensity conditioning
RTC: Reduced-toxicity conditioning
STR: Short tandem repeat
TBI: Total body irradiation
XHIGM: X-linked hyper-IgM syndrome
