# Supplementary material for: Allogeneic hematopoietic cell transplantation with reduced-toxicity conditioning for X-linked hyper-IgM syndrome
Source: Int J Hematol. 2026 Jul 7;124(5):704–12. doi: 10.1007/s12185-026-04245-8 (PMC13719731; doi:10.1007/s12185-026-04245-8)
Supplement: Supplementary file 1 [file 12185_2026_4245_MOESM1_ESM.docx]

Supplementary materials for "**Allogeneic hematopoietic cell transplantation with reduced toxicity conditioning for X-linked hyper-IgM syndrome**" in International Journal of Hematology

Ryosuke Wakatsuki, Satoshi Miyamoto, Akihiro Hoshino, Shuhei Takahashi, Yusuke Ishibashi, Ryohei Watanabe, Tatsuya Magara, Dan Tomomasa, Akira Nishimura, Kyogo Suzuki, Takahiro Kamiya, Takeshi Isoda, Kohsuke Imai, Michiko Kajiwara, Tomohiro Morio, Masatoshi Takagi, Hirokazu Kanegane

Corresponding author

Hirokazu Kanegane, M.D., Ph.D.

Department of Child Health and Development, Graduate School of Medical and Dental Sciences, Institute of Science Tokyo

1-5-45 Yushima, Bunkyo-ku, Tokyo 113-8519, Japan

E-mail: hkanegane.ped@tmd.ac.jp

**Supplementary Figure 1. HLA-based flow cytometry (HLA-flow)**

(A) HLA-flow results of patient 2 on post-transplant day 1001. Donor-derived cells account for 94% of the total population.

(B) HLA-flow results of patient 6 on post-transplant day 34. Donor-derived cells account for 2.77% of the total population.

**Supplementary Figure 2. Chimerism assessment using droplet digital polymerase chain reaction (ddPCR)**

(A) Results of sex-mismatched ddPCR using *SRY* for patient 8 on post-transplant day 603. In whole blood, 24.2% of cells are donor-derived.

(B) Results of variant-specific ddPCR for patient 9 on post-transplant day 43. In whole blood, 97.4% of cells are donor-derived.

(C) Results of variant-specific ddPCR for patient 10 on post-transplant day 252. In whole blood, 95.8% of cells are donor-derived.

WB: whole blood, Gr: granulocytes, MNC: monocytes, CD3+: CD3-positive T cells, CD19+: CD19-positive B cells.

**Supplementary Figure 3. Discontinuation rate of immunoglobulin replacement therapy**

The discontinuation rate for immunoglobulin replacement therapy (IgRT) is reported for 8 of the 9 surviving patients, excluding patient 6 for whom the clinical course of IgRT could not be assessed. During the follow-up period, only patient 8 continued IgRT.

**Supplementary Figure 4. Longitudinal changes in whole-blood and T-cell chimerism assessed by droplet digital PCR**

Longitudinal changes in whole-blood and T-cell chimerism assessed by droplet digital PCR are shown for three patients. Panels indicate (A) patient 8, (B) patient 9, and (C) patient 10.
